# Lead Poisoning Revealed by Unexplained Abdominal Pain and Anemia in a Young Adult: A Diagnostic Challenge

**DOI:** 10.1002/ccr3.72172

**Published:** 2026-02-28

**Authors:** Jennifer Eichler, Sarah Albrecht, Lars C. Huber

**Affiliations:** ^1^ Department of Internal Medicine Stadtspital Zurich Zurich Switzerland; ^2^ Department of Internal Medicine University Hospital Zurich and University of Zurich Zurich Switzerland

**Keywords:** abdominal pain, anemia, basophilic stippling, Burton's line, chelation therapy, DMSA, lead poisoning

## Abstract

Lead poisoning should be considered in patients presenting with unexplained anemia and abdominal pain, even without clear environmental or occupational exposure. Recognizing classic signs such as Burton's line and basophilic stippling enables timely diagnosis and effective chelation therapy.

## 1 Introduction

Lead poisoning remains an important differential diagnosis in patients presenting with unexplained abdominal pain and anemia, despite its declining incidence. Its nonspecific clinical presentation often delays diagnosis. We present a case of lead intoxication in a young adult, highlighting the importance of classical diagnostic signs, such as Burton's line and basophilic stippling, in facilitating timely recognition and management.

## 2 Case Presentation

A 29‐year‐old man with no significant medical history presented to the emergency department with acute abdominal pain and intermittent vomiting lasting 1 week. He reported progressive fatigue and generalized weakness over the preceding 3 months. He denied recent travel, medication use, or exposure to environmental toxins.

On examination, the patient was afebrile, with a severely elevated blood pressure of 205/110 mmHg, heart rate of 100 bpm, and normal oxygen saturation while breathing ambient air. He appeared ill and disoriented. Abdominal examination revealed diffuse tenderness with preserved bowel sounds and no peritoneal signs. A linear bluish discoloration along the gingival margin was observed, suggestive of a possible Burton's line. Given its recent onset and location, differentiation from early dental changes cannot be definitively excluded. The remainder of the examination was unremarkable.

## 3 Investigations

**FIGURE 1:**
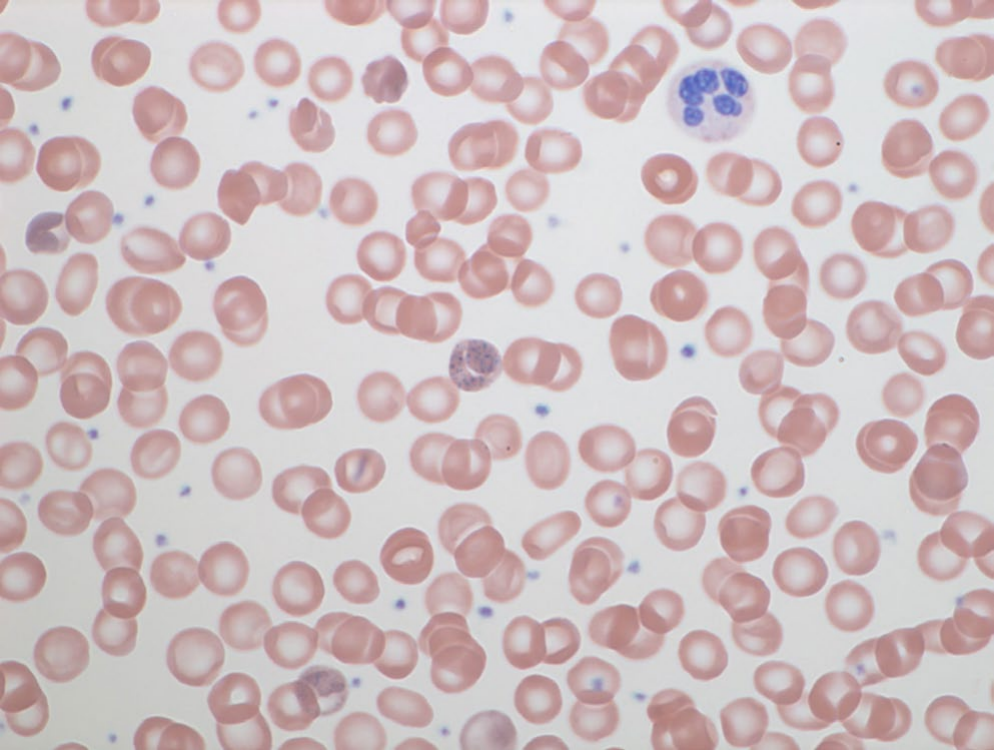
Basophilic stippling of erythrocytes on peripheral blood smear, indicative of disturbed erythropoiesis.

Laboratory findings are summarized in Table 1 and revealed normocytic, normochromic anemia with reticulocytosis. Total bilirubin was elevated, predominantly because of the indirect fraction, consistent with hemolysis. Lactate dehydrogenase remained within the normal range on serial measurements. Haptoglobin was not determined, as acute abdominal pain predominated and the initial evaluation focused on erythropoietic substrates, including ferritin, folate, and vitamin B12, all of which were within normal limits. Peripheral blood smear demonstrated coarse basophilic stippling of erythrocytes (Figure 1). Abdominal ultrasound showed no hepatobiliary disease or gallstones. Abdominal CT was performed because of the severity of abdominal pain to exclude bowel obstruction or malignancy. Given the patient's young age, previously good health, and the degree of coprostasis observed on CT, upper and lower endoscopy were subsequently performed to exclude malignant lesions and chronic inflammatory bowel disease. Spot urine testing for porphobilinogen and aminolevulinic acid (ALA) was initially normal; however, quantitative analysis revealed an elevated ALA level without corresponding increase in porphobilinogen. These findings raised suspicion for a heavy metal–induced disruption of heme synthesis. Subsequent testing confirmed a significantly elevated blood lead level (3.22 μmol/L; reference < 1.93 μmol/L), establishing the diagnosis of lead intoxication. Differential diagnoses included gastrointestinal inflammatory disorders, acute porphyria, iron deficiency anemia, thalassemia, and general intoxication.

**TABLE 1:** Selected laboratory results at presentation.

Parameter	Result	Reference range	Interpretation
Hemoglobin	11.4 g/dL	13.5–17.2	↓ Mild anemia
MCV	89 fL	80–96	Normal
WBC	11.9 × 10^9^/L	4.0–10.0	↑ Mild leukocytosis
Platelets	245 × 10^9^/L	150–400	Normal
Reticulocytes	3.82%	0.5–2.0	↑ Elevated
Total bilirubin	33 μmol/L	< 21	↑ Indirect fraction
AST/ALT	Normal	< 35 U/L	—
CRP	Normal	< 0.5 mg/dL	No inflammation
Creatinine	0.74 mg/dL	0.74–1.35	Normal

Abbreviations: ALT, alanine transaminase; AST, aspartate transaminase; CRP, C‐reactive protein; MCV, mean corpuscular volume; WBC, white blood cells.

## 4 Treatment

Tox Info Suisse, the national poison information center, was consulted. In light of severe, opioid‐refractory abdominal pain compatible with lead colic and moderately elevated blood lead levels, oral dimercaptosuccinic acid (DMSA) therapy (30 mg/kg/day for 5 days, then 20 mg/kg/day for 14 days) was recommended. The regimen was well tolerated; no adverse effects were noted. Within 48 h, abdominal pain subsided, appetite improved, and lethargy regressed. Serial monitoring demonstrated a progressive decline in blood lead concentration and normalization of hemoglobin and reticulocyte counts over several weeks.

No public health notification was made, as this was an isolated case with no similar symptoms in the patient's contacts, and lead intoxication is not a notifiable condition in Switzerland.

## 5 Outcome and Follow‐Up

The patient's clinical condition improved continuously. At 3‐month follow‐up, he remained asymptomatic, with normal renal function and no neurological deficits. Blood lead levels declined from 3.22 μmol/L at presentation to 1.15 μmol/L at completion of chelation therapy and further to 0.47 μmol/L 1 year later. Mild elevations can persist because of continued mobilization of lead from tissue stores, even after clinical recovery. He was counseled to avoid further use of lead‐containing ceramics and educated about potential environmental sources of heavy metals.

## 6 Discussion

Lead poisoning, though rare in industrialized countries, remains a significant differential diagnosis in patients presenting with unexplained anemia and abdominal pain. Non‐occupational exposures such as traditional medicines [1], contaminated ceramics [2], or substance abuse [3] are recognized sources of intoxication. Clinical manifestations are often nonspecific and may include fatigue, nausea, constipation, and neuropsychiatric symptoms. Classic signs such as Burton's line and basophilic stippling of erythrocytes, when present, represent valuable diagnostic clues. However, the linear blue‐purplish discoloration at the junction between the teeth and gums (Burton's line) may be subtle and can be confounded by local dental changes.

Lead disrupts heme biosynthesis by inhibiting key enzymes within this pathway [4], most notably δ‐aminolevulinic acid dehydratase (ALAD) and ferrochelatase. Inhibition of ALAD leads to accumulation of aminolevulinic acid (ALA) without a corresponding increase in porphobilinogen, thereby distinguishing lead toxicity from acute porphyria. Inhibition of ferrochelatase prevents incorporation of iron into protoporphyrin IX, resulting in zinc protoporphyrin formation and contributing to anemia.

Additionally, lead exposure impairs erythrocyte pyrimidine 5′‐nucleotidase activity [5], leading to accumulation of pyrimidine nucleotides and the characteristic basophilic stippling observed on peripheral blood smears. Although microcytic anemia is classically associated with chronic lead exposure, normocytic anemia has also been described in adults, particularly in cases of acute or subacute exposure [6]. Similarly, the acute onset of abdominal pain observed in this patient contrasts with the more typically chronic symptomatology of lead intoxication.

The patient worked in an office setting and reported no contact with typical occupational or environmental lead sources. Further history revealed that he had recently moved into a shared apartment and was the only resident who drank daily from a glazed ceramic teapot. According to the patient, the teapot originated from Turkey. This domestic use is retrospectively suspected to be the source of lead exposure.

Environmental and lifestyle factors should therefore be carefully considered even in non‐occupational settings. In this case, domestic use of glazed ceramics represents the most likely source, although other exposures—including older buildings, contaminated water systems, or hobbies involving lead‐containing materials—should also be evaluated.

The nonspecific clinical presentation complicates diagnosis and often delays recognition. Iron deficiency anemia, thalassemia, gastrointestinal disorders, and acute porphyria [7] were excluded through targeted laboratory investigations and imaging studies. A thorough exposure history combined with focused diagnostic testing was essential for establishing the correct diagnosis.

This case highlights significant lead intoxication in a young adult without occupational exposure, presumably caused by domestic use of glazed ceramics—an underrecognized environmental source. It underscores the importance of considering environmental and lifestyle factors when evaluating unexplained anemia and abdominal pain. Early identification and removal of the exposure source, together with timely chelation therapy, are crucial to prevent serious complications, including neurological and renal damage.

## 7 Conclusions

Lead intoxication should be suspected in patients with unexplained abdominal pain and anemia, even without occupational exposure. This case highlights the preventable nature of lead poisoning and underscores its relevance as a public health issue, even in industrialized settings. Early recognition and source identification are essential to avoid long‐term complications.

## 8 Learning Points

- Lead poisoning remains a diagnostic challenge because of nonspecific symptoms.
- Presence of Burton's line and basophilic stippling should prompt lead testing.
- Environmental sources such as glazed ceramics or lead‐based materials in older housing [8] are underrecognized.
- DMSA chelation therapy is effective and well‐tolerated for moderate intoxication.

## Author Contributions

**Jennifer Eichler:** conceptualization, data curation, investigation, writing – original draft. **Sarah Albrecht:** data curation, investigation, writing – review and editing. **Lars C. Huber:** conceptualization, supervision, writing – review and editing.

## Funding

The authors have nothing to report.

## Consent

Written informed consent was obtained from the patient for the publication of this case report, including all clinical information and images.

## Conflicts of Interest

The authors declare no conflicts of interest.

## Data Availability

Due to patient privacy and confidentiality, the data supporting this case report are not publicly available.

## References

[1] C. Ciocan , I. Mansour , A. Beneduce , et al., “Lead Poisoning From Ayurvedic Treatment: A Further Case,” La Medicina del Lavoro 112, no. 2 (2021): 162–167, 10.23749/mdl.v112i2.10576. 33881010 PMC8095331

[2] M. Mohamed , A. Ugarte‐Torres , H. Groshaus , K. Rioux , and M. Yarema , “Lead Poisoning From a Ceramic Jug Presenting as Recurrent Abdominal Pain and Jaundice,” ACG Case Reports Journal 3, no. 2 (2016): 141–143, 10.14309/crj.2016.27. 26958573 PMC4748209

[3] M. Abedi Samakoosh , A. Sharifpour , M. Soleymani , and Z. Zakariaei , “Lead Poisoning With Unusual Manifestations in an Opium‐Addicted Man,” Clinical Case Reports 10, no. 12 (2022): e6774, 10.1002/ccr3.6774. 36590665 PMC9794920

[4] M. M. Diawara , “Pharmacogenetics of Lead Toxicity,” Medical Research Archives 13, no. 1 (2025): 6268, 10.18103/mra.v13i1.6268.

[5] E. Bitto , C. A. Bingman , G. E. Wesenberg , J. G. McCoy , and G. N. Phillips, Jr. , “Structure of Pyrimidine 5′‐Nucleotidase Type 1,” Journal of Biological Chemistry 281, no. 29 (2006): 20521–20529, 10.1074/jbc.M602000200. 16672222

[6] B. Pumallanqui‐Ramirez , J. Li , L. Torres , and J. Rosales‐Rimache , “Relationship Between Blood Lead Levels and Anemia: A Cross‐Sectional Study on Mining Workers From Peru,” Advances in Public Health 2024 (2024): 5517405, 10.1155/2024/5517405.

[7] M. T. Tsai , S. Y. Huang , and S. Y. Cheng , “Lead Poisoning Can Be Misdiagnosed as Acute Porphyria,” Case Reports in Emergency Medicine 2017 (2017): 9050713, 10.1155/2017/9050713. 28630774 PMC5467293

[8] T. D. Sowers , C. M. Nelson , M. D. Blackmon , et al., “United States House Dust pb Concentrations Are Influenced by Soil, Paint, and House Age: Insights From a National Survey,” Journal of Exposure Science & Environmental Epidemiology 34, no. 4 (2024): 709–717, 10.1038/s41370-024-00655-0. 38548929 PMC11303246

